# Plugging the drain: targeted policies for retaining Israeli doctors

**DOI:** 10.1186/s13584-025-00737-w

**Published:** 2025-11-28

**Authors:** Yoel Angel, Nevo Barel, Gil Fire

**Affiliations:** 1 Tel Aviv Sourasky Medical Center, Tel Aviv, Israel; 2 The Gray School of Medicine, Coller School of Management, Tel Aviv University, Tel Aviv, Israel

**Keywords:** Physician shortage, Foreign medical graduates, Healthcare policy

## Abstract

Israel’s physician workforce crisis reflects long-standing dependence on foreign-trained doctors, limited domestic capacity, and the shrinking inflow of new graduates following the Yatziv Reform. Recent analyses underscore the need not only to expand local training but also to better track and support Israelis studying and training abroad. While new data such as that by Swechinsky and Berner-Shalem shed light on return patterns among students, a less visible challenge lies with physicians leaving the Israeli health system, and specifically those undertaking fellowship training overseas who choose not to return. Building reliable data systems and proactive engagement mechanisms for both students and fellows is essential to securing Israel’s future medical workforce.

In recent years, an emerging crisis in Israel’s physician workforce has drawn growing attention from policymakers, researchers, and health system leaders [1, 2]. This crisis stems from both a fragile structural foundation and the convergence of several external shocks. The fragility of the system is evident in Israel’s heavy reliance on foreign-trained physicians- the highest in the OECD by far, with nearly 60% of new physicians educated abroad [3] -alongside limited domestic medical school capacity and the absence (until recently) of a long-term national workforce strategy.

Three major developments have further exacerbated this situation: the retirement of a large cohort of senior physicians, the sharp decline in the number of Israeli students graduating from non-recognized foreign medical schools following the Yatziv Reform, and continued population growth with rising healthcare needs. In response, the Ministry of Health (MoH), particularly its Division for Strategic Planning, initiated a series of comprehensive workforce analyses and policy interventions. These included a dedicated OECD review [4], the first and second Gamzu Committees focusing on health workforce and medical education [5, 6], and a range of initiatives designed to expand medical school capacity, strengthen clinical training, and promote equitable geographic distribution of physicians, particularly in peripheral regions.

Many of these initiatives are already yielding tangible results. Most prominently, the number of students beginning medical school in Israel has doubled-from 728 in 2018 to 1448 in 2025 [7]. At the same time, recognition has grown that Israeli students studying medicine abroad remain an essential component of the national health workforce pipeline. Accordingly, the MoH launched several targeted programs, including a voluntary registry for medical students abroad, collaborations with student associations, incentives for Israeli hospitals to host visiting clinical rotations, and the Ofakim program, which provides financial support to returning graduates. Nevertheless, persistent gaps in reliable data on Israelis studying medicine overseas continue to hinder effective planning and policy implementation.

Within this context, we commend Swechinsky and Berner-Shalem on their important and innovative approach to data collection, which relied on cross-referencing voluntary reports from Israeli students abroad [8]. This is the first structured, multi-country examination of return behavior among Israeli medical graduates trained abroad. Across 37 classes comprising approximately 1,048 graduates in 11 countries, the reported non-return rate was 9.7% (12.1% when excluding Palestinian Authority institutions), with substantial heterogeneity by country (close to 0% in Jordan vs. about 40% in Italy). Larger Israeli class size was correlated with lower non-return, while higher host-country Human Development Index (HDI) was associated with higher rates of non-return. These findings move the discussion away from a simplistic, uniform notion of “brain drain” and toward recognition of specific, context-dependent risk factors that require targeted policy responses.

The study’s innovative methodology incurs some inherent limitations, also acknowledged by the authors, which constrain the strength of the conclusions. The data are voluntary, self reported, and not validated against objective sources such as immigration or fiscal records; they also contain inconsistencies and incomplete coverage, such as the relatively wide interquartile range of class sizes in some institutions. As such, they should be regarded as indicative rather than definitive. The reported associations should likewise not be interpreted as causal. Thus, although this work represents an important first step, the findings warrant cautious interpretation and should be taken with a grain of salt.

The finding of a lower than anticipated non-return rate among students abroad should not lead to complacency. Instead, it highlights where scarce policy resources might best be directed: toward specific countries and faculties, and toward developing more effective return pathways, while in parallel continuing to expand domestic training reforms. The considerable heterogeneity described by Swechinsky and Berner-Shalem underscores the need for tailored interventions rather than a uniform approach. Such measures should be adapted to the circumstances of each country and medical school, prioritizing engagement with faculties and cohorts that show higher non return rates, strengthening structured return pathways in settings with strong pull factors, and examining whether supporting study within established Israeli cohorts abroad may indirectly enhance return while maintaining educational quality.

Recognizing that even after the Yatziv reform, Israel will still rely heavily on foreign medical graduates, this study underscores the urgent national need for more robust and comprehensive data on Israelis studying abroad and their return rates. Potential strategies include cross linkage of administrative sources such as border control records and taxation data; creation of structured incentives, both positive and negative, for accurate self reporting of study abroad and return intentions; and direct engagement with high yield faculties through on site surveys to identify Israeli students. Such steps are essential for long term workforce planning and will not be achieved without deliberate investment. Importantly, these approaches could later be adapted for other professional groups training abroad, both within and outside the health sector.

The multitude of interventions that have focused on increasing the entry of new physicians into the Israeli health system is noteworthy. In contrast, far less attention has been given to the retention of the existing workforce, also a significant factor in determining size of the workforce. The loss of an specialist physician following years of experience in the Israeli system is no less, and perhaps even more influential, than the non-return of a foreign medical graduate, due to their substantial contribution to the system’s capacity and their position in training of domestic students and residents. Nonetheless, losses continue to occur both through internal career changes-often linked to financial considerations or burnout, a concern in Israel as elsewhere-and through outright emigration. The latter appears increasingly salient in recent years, amid social unrest related to judicial reform and, more recently, the consequences of the October 7 war, with anecdotal yet widely discussed accounts of more physicians leaving Israel. Reliable estimates of such cases are not currently available, although they should be relatively straightforward to identify through national databases like border and taxation data.

A more elusive challenge is monitoring Israeli physicians who undertake fellowship training abroad and do not return. Such fellowships are a natural step in the career trajectory of many young specialists, yet they create a potential loophole in retention. Israeli clinicians are highly regarded internationally and often receive offers to remain abroad. This presents a significant retention challenge for a small health system such as Israel. Furthermore, many fellowships are arranged independently and at considerable personal and financial cost, leaving physicians effectively outside the Israeli system. This can foster feelings of isolation or alienation, further reducing the likelihood of return.

Unlike medical students, for whom partial data exist thanks to initiatives like those of Swechinsky and Berner-Shalem, there are currently no reliable national data on the number of Israeli physicians undertaking fellowship training abroad or on their eventual return. Definitions further complicate measurement: is a second or third “additional year” still part of training, or the first step toward permanent emigration? Addressing this gap requires development of clear definitions and a longitudinal database that captures these movements over time and enables the identification of emerging trends.

Several initiatives are underway to address these challenges. One is the work of non profit organizations such as Science Abroad, which supports and networks Israeli scientists and physicians overseas facilitating their return to Israel. Another example are programs like that at Tel Aviv Sourasky Medical Center (Ichilov), where a dedicated interdepartmental task force was established to identify, track, and support physicians training abroad. Early audits revealed significant undercounting of fellows overseas. The task force now maintains proactive contact throughout training, provides support, and keeps a live roster to facilitate timely return. The rationale is clear: maintaining connection and visibility increases the likelihood of return. This hospital level model illustrates an approach that could be replicated by other centers and standardized nationally.

## Policy recommendations

To address these challenges, we advocate for further policy steps to be taken: First, we support the recommendation of Swechinsky and Berner-Shalem: the existing voluntary registry of Israeli medical students abroad should be expanded and formalized through well-designed incentives to improve completeness and accuracy. We call for it to also encompass Israeli physicians engaged in postgraduate or fellowship training overseas as a critical first step to start adressing this underrecognized population. Second, data from administrative sources such as immigration records, census information, taxation files, and other relevant datasets should be analyzed to create a more comprehensive national view and identify individuals who have not voluntarily registered. Third, periodic field audits should be conducted at high-yield locations (“boots on the ground”) - particularly where large Israeli cohorts or higher risks of non-return exist-to verify numbers, host informational sessions, and establish structured return pathways.Fourth, maintaining structured, proactive contact with registrants is essential; regular communication should be used to validate information, update intentions regarding return, and reinforce affiliation with the Israeli health system. The latter recommendations may be augmented by working closely with local Israeli embassies to serve as a designated point of contact for, and proactively reach out to, Israeli medical students and physicians abroad. Lastly, we must ensure that returning physicians and physician-scientists have appropriate opportunities awaiting them in Israel. For clinicians, this means guaranteeing sufficient open resident and attending positions with clear employment pathways upon return. For physician-scientists, it also requires funding, infrastructure and institutional support to enable them to continue their academic and scientific work in Israel.We believe such steps may have a substantial contribution to the Israeli health workforce and may serve as a model for other countries to follow, and call on the Israeli government embark on these steps with sufficient allocation of resources to ensure their success.

## Conclusion

While retention of health workforce is a global challenge, Israel faces unique demographic and structural difficulties in this field. The preliminary data presented by Swechinsky and Berner-Shalem offer valuable insights into the return intentions of Israelis studying abroad, but mostly highlight the need for more and better information on this population, as well as on other populations of Israelis training abroad, particularly during fellowship training. Beyond information, significant resources and attention must be directed towards these populations in order to influence their return patterns.

## Data Availability

Not applicable.
